# Self-harm in the cycle of (moral) emotions: an exploratory analysis of emotional triggers and responses

**DOI:** 10.3389/fpsyg.2026.1783881

**Published:** 2026-10-28

**Authors:** Andrej Demuth, Slavka Demuthova, Rebeka Demuthova

**Affiliations:** 1 Department of Theory of Law and Philosophy of Law, Comenius University in Bratislava, Bratislava, Slovakia; 2 Department of Psychology, University of Ss. Cyril and Methodius in Trnava, Trnava, Slovakia; 3 School of Biomolecular and Biomedical Science, University College Dublin, Dublin, Ireland; 4 Department of Human Genetics, Faculty of Medicine and Health Sciences, McGill University, Montreal, QC, Canada

**Keywords:** emotions, gender, religious identification, self-harm, young adults

## Abstract

Self-harm represents a serious mental health concern in which emotional experiences play a significant role in both its onset and maintenance. This exploratory study examined which emotions were most frequently reported before and after self-harm, how selected emotions changed following self-harm, and how emotional experiences were associated with specific forms of self-harm and demographic factors (gender, religious identification). The sample comprised of 158 university students, of whom 130 reported a history of self-harming behavior and were included in analyses of emotional experiences. Data were collected using a modified Self-Harm Inventory questionnaire and measures assessing the occurrence of emotions before and after self-harm. Anger, feelings of failure, and sadness were the most frequently reported emotions preceding self-harm, whereas sadness, relief, disappointment, guilt, and shame were the most frequently reported emotions following self-harm. The reported frequency of anger, guilt, and sadness decreased following self-harm, whereas negative emotions remained prominent after the behavior. After Benjamini–Hochberg correction for multiple comparisons, religious identification remained negatively associated with self-harm frequency and with the reported frequency of several emotions. Gender was not significantly associated with the examined variables, although this finding should be interpreted cautiously given the gender imbalance and limited sample size. After correction, selected associations between emotions and specific forms of self-harm remained significant, including associations of anger with hitting oneself, disgust with starving oneself, hatred with self-defeating thoughts, guilt following laxative misuse, and changes in anger with hitting oneself and intentionally worsening medical situations. Overall, the findings indicate selective associations between emotional experiences and self-harming behavior while underscoring the need for longitudinal and event-level research to clarify their temporal and causal dynamics.

## 1 Introduction

Self-harm represents a serious and growing public health problem (Wu et al., 2025), the consequences of which extend beyond the individual psychological level to affect social functioning (disruption of relationships—Hasking et al., 2015; stigmatization—Meheli and Banerjee, 2022; educational consequences—Baetens et al., 2021) as well as the economic domain (Kinchin et al., 2017; Peterson et al., 2024). Self-harm significantly reduces quality of life and is also associated with a shortened life expectancy (by an average of 26 years) (Bergen et al., 2012), even in the absence of suicidal intent. Empirical studies indicate a high prevalence of self-harm (Muehlenkamp et al., 2012; Swannell et al., 2014), in some contexts reaching an epidemic level (Molchanova, 2024), particularly among adolescents and young adults (Apicella et al., 2025). Occurrence has been documented across cultures, with higher rates reported in Asia (Xiao et al., 2022), more frequently among girls and young women (with the exception of Asia—Moloney et al., 2024), showing an increasing trend (Mitchell et al., 2023; Liu et al., 2023) and a decreasing age of onset (Gandhi et al., 2018; Wångby-Lundh et al., 2023).

One of the fundamental challenges in self-harm research is the absence of a universally accepted and consensual definition. The Diagnostic and Statistical Manual of Mental Disorders, Fifth Edition (DSM-5) proposes very narrow term “Non-Suicidal Self-Injury” (American Psychiatric Association, 2013), focusing primarily on direct physical forms. The International Classification of Diseases, 11th Revision (ICD-11) expands the conceptualization of self-harm within the category “Intentional self-harm” to include additional indirect and covert forms (World Health Organization, 2022), yet it still primarily addresses damage to somatic health. However, several authors emphasize that the spectrum of self-harming behavior is substantially broader (Sansone and Sansone, 2010; Hooley et al., 2020; Liljedahl et al., 2023), including behaviors that primarily damage an individual’s psychological well-being and are aimed not at physical but at psychological suffering (Demuthova, 2023; Liljedahl et al., 2023). A common denominator across different conceptual approaches to self-harm is the intentional and deliberate infliction of pain or harm as the primary goal of the behavior. This criterion allows self-harm to be distinguished from accidental injuries, behaviors lacking full awareness, or activities in which pain is not the intended outcome (e.g., tattooing).

In the present study, self-harm is understood as a conscious and intentional infliction of subjectively perceived harm (physical, mental, social, or spiritual), typically accompanied by pain (somatic and/or psychological). This is, therefore, a definition that is broader than the one provided in the DSM-5 and includes (besides behaviors included within non-suicidal self-injury) also various forms of hidden physical self-harm (ICD-11), and also mental self-harm (Sansone and Sansone, 2010) including suicidal tendencies, which have been shown to be a part of self-harming behavior in many studies (see, e.g., Hawton et al., 2012; Grandclerc et al., 2016; Bettis et al., 2020). Our study, however, did not assess self-harm as a clinical category (which—besides others—needs to fulfill also other criteria), but assessed the self-reported lifetime occurrence of any form of self-harming behavior based on this wide definition.

Among the most frequently reported reasons for self-harm are intrapersonal factors, particularly the regulation of negative emotions (Klonsky, 2007; Nock, 2010), self-punishment (Glassman et al., 2007), antidissociation (Briere and Gil, 1998; Simeon and Favazza, 2001), and the attainment of a sense of control or relief (Chen et al., 2025; Wu et al., 2025). Emotions, in particular, emerge as a highly salient component not only in the antecedents and motives of self-harm but also in the intervening factors and mechanisms that reinforce and maintain self-harm within the repertoire of risky and maladaptive behaviors. In recent years, special attention has been devoted to moral emotions. This concept, first defined by Haidt (2003), refers to emotions related to the moral evaluation of one’s own or others’ behavior in relation to norms, values, expectations, and conscience. In our study moral emotions are understood as affective states associated with ethical evaluation and with the appraisal of oneself, others, actions, or situations in relation to moral values and norms (Demuth and Demuthova, 2023).

Within the present study, anger and disgust are treated as potentially other-condemning moral emotions, whereas guilt and shame are treated as self-conscious moral emotions when they occur in an evaluative context involving norms, values, or perceived wrongdoing. However, anger, sadness, and disgust are not intrinsically moral in every instance, and the questionnaire did not establish whether each reported emotional episode involved an explicit moral evaluation or perceived norm violation. The study therefore distinguishes emotions commonly understood as moral in relevant evaluative contexts from other emotional states associated with self-harm.

Research examining the relationship between moral emotions and self-harm remains relatively scarce. Among the existing studies is the work of Bryan et al. (2014), which found that emotions related to moral failure, unworthiness, and impurity (moral injury) are frequent antecedents of self-harm both among soldiers who have engaged in morally unacceptable behavior and among victims, particularly following trauma related to sexual abuse (Talbot et al., 2004). According to Cheng et al. (2025), self-hate mediates the relationship between adverse childhood experiences and self-harm in adolescents. Similarly, Brown et al. (2009) reported that high scores on measures of shame and guilt are strongly correlated with the frequency and severity of self-harm. Xavier et al. (2015) identified internalized and non-verbalized feelings of shame and guilt as frequent triggers not only of self-harm but also of suicidal behavior.

A repeatedly confirmed finding is that tendencies toward internalization are particularly characteristic of the female gender (Gutman and Codiroli McMaster, 2020; González-Rubio et al., 2023). Girls experience emotional problems internally to a much greater extent than boys (Babicka-Wirkus et al., 2023) and more frequently suffer from depression, anxiety, thought problems, and withdrawal (González-Rubio et al., 2023). In contrast, boys tend toward externalization (with an average male-to-female ratio of 2.5:1—Hicks et al., 2007), expressing emotions outwardly and addressing problems through behaviors that may result in aggressive or delinquent conduct (González-Rubio et al., 2023). These findings support the assumption that gender may also play a key role in the emotional patterns accompanying self-harm (e.g., internalized guilt versus externalized anger). An interesting study by Tong and Teo (2018) examined the influence of religious concepts on the effects of blame appraisals on negative emotions such as anger or guilt. Several experiments demonstrated that thoughts of God can reduce anger but neither mitigate nor intensify guilt. These findings suggest that religiosity meaningfully intervenes in the experience of moral emotions and should therefore be considered in research.

In the context of examining the role of moral emotions in the process of self-harm, the so-called cyclical hypothesis of self-harm appears particularly informative (Sutton, 2007). This model describes a process in which emotional suffering leads to self-harm as a source of short-term relief, followed by feelings of shame and guilt, thereby reinitiating the cycle. The model emphasizes the importance of examining changes in the frequency of occurrence of emotions before and after self-harm and their relationship to specific forms of self-harm. Therefore, research in this area has the potential not only to provide missing data on the experience of (moral) emotions associated with self-harm but also to contribute empirical evidence potentially supporting the cyclical hypothesis of self-harm. Important factors that should be included in analyses of self-harm, its forms, and associated emotions are gender (Gutman and Codiroli McMaster, 2020; Moloney et al., 2024) and religiosity (Sheridan et al., 2011; Tong and Teo, 2018).

Given the lack of research in this area, an exploratory research design was employed with the primary aim of identifying the occurrence and role of selected emotions in self-harm, specifically to clarify the relationships between self-harm (and its forms), emotions (before self-harm, after self-harm, and changes in the frequency of their occurrence), and selected demographic factors (gender, religious identification). In line with this aim, the following research questions were formulated:

Research Question 1: Is the reported frequency of self-harming behavior associated with demographic factors? (gender, religious identification).
Research Question 2: Which emotions most frequently precede self-harming behavior, and which emotions most commonly follow self-harming behavior?
Research Question 3: Are demographic factors (gender, religious identification) associated with the frequency of occurrence of emotions before and after self-harm?
Research Question 4: For which emotions (anger, guilt, shame, and sadness) did the frequency of occurrence change significantly after self-harm compared to the pre–self-harm state?
Research Question 5: Are demographic factors (gender, religious identification) associated with the magnitude of change in the frequency of occurrence of emotions (anger, guilt, shame, and sadness) before and after self-harm?
Research Question 6: Is there a relationship between (a) the frequency of occurrence of emotions before self-harm and the form of self-harm; (b) the frequency of occurrence of emotions after self-harm and the form of self-harm; and (c) the magnitude of change in the frequency of occurrence of emotions (anger, guilt, shame, and sadness) and the form of self-harm?
Research Question 7: Accounting for gender, and the different frequencies of self-harm, does religious identification remain associated with the emotions before and after self-harm, and with changes in the pairwise emotions?

## 2 Materials and methods

### 2.1 Procedure and research sample

Data collection took place between October 2024 and March 2025 at the Faculty of Arts of St. Cyril and Methodius University in Trnava and at the Faculty of Law of Comenius University in Bratislava (Slovakia). 165 participants were recruited through convenience sampling and included full-time psychology students (*N* = 96), part-time psychology students (*N* = 46), and full-time law students (*N* = 23). During regular classes, students were informed about the nature and aims of the study, and to ensure anonymity, they were subsequently provided with a QR code enabling electronic completion of the questionnaire via an online form in the Forms application. Non-participation in the study or withdrawal at any point during the procedure resulted in no negative consequences for the participants. Five participants were excluded from the final analysis because they did not consent to the use of their data. Two additional participants were excluded due to internally inconsistent questionnaire responses (specifically reporting no history of self-harm while simultaneously reporting emotions associated with the periods before and after self-harming behavior).

The research sample consisted of 158 participants, of whom 134 (84.81%) were women. Fifteen participants were younger than 19; seventy-one were aged 19–20, and the remaining 72 students were older than 20 years. Twenty-eight participants did not report any form of self-harming behavior; these participants were excluded from the analyses addressing research questions Q2–Q7. A total of 130 participants were included in final analyses, of whom 84.62% were women and 15.38% men; for responses “other” and “prefer not to disclose” *N* = 0. 83 participants (63.85%) stated they were believers, 15 (11.54%) were undecided, and the remaining 32 participants (24.61%) identified as non-believers. For “prefer not to disclose” *N* = 0.

### 2.2 Measures

Data were collected using an online questionnaire consisting of three sections. The first section focused on informed consent and the collection of demographic data on gender and religious identification. For the assessment of gender, participants were provided with four response options: man (0), woman (1), other (2), and prefer not to disclose (3). Regarding religious identification, participants were likewise provided with four response options: non-religious (0), unsure (1), religious (2), and prefer not to disclose (3).

The second section assessed (1) the presence of self-harming behavior and (2) the reported frequency of twenty specific forms of intentional self-harming behavior. For this purpose, a modified version of the Self-Harm Inventory (SHI; Sansone and Sansone, 2010) was used. The modification was implemented in two ways: (1) based on extensive research mapping the prevalence of self-harming behaviors in the Slovak population (see Demuthova, 2023), four items from the original SHI were removed (Lost a job on purpose, Engaged in sexually abusive relationships, Driven recklessly on purpose, and Been promiscuous) and replaced with more frequently occurring forms of self-harm (Not slept enough to hurt yourself; Over-exercised to hurt yourself); and (2) the presence of each of the twenty forms of self-harm was additionally assessed in terms of frequency using a forced-choice Likert-type scale: never (0), rarely (0.25), sometimes (0.5), often (0.75), and almost always (1). These values represent exploratory numerical codes for the occurrence of individual forms of behavior (exploratory operational categories of frequency), which were used to quantify responses for the purposes of statistical analysis in this exploratory study. They should not be interpreted as interval-level measures of severity, intensity, or clinical risk of self-harm. The wording of the modified SHI questionnaire and the list of assessed forms are presented in Table 1. The modified SHI demonstrated good internal consistency (Cronbach’s alpha = 0.877). No validated clinical cut-offs were applied, and the reported proportions are interpreted only as descriptive frequencies within the present operational framework.

**Table 1:** Forms of self-harm assessed using the modified self-harm inventory and the frequency of their occurrence in the sample (*N* = 158).

Have you ever intentionally, or on purpose, done any of the following			%
Behavior:	%	Behavior	
Tortured yourself with self-defeating thoughts	51.3**	Engaged in emotionally abusive relationships	32.9
Scratched yourself on purpose	44.9	Starved yourself to hurt yourself	38.6
Hit yourself	47.5	Prevented wounds from healing	7.6*
Cut yourself on purpose	24.1	Burned yourself on purpose	6.3
Exercised an injury on purpose	24.8*	Attempted suicide	9.5
Banged your head on purpose	22.8	Distanced yourself from God as a punishment	9.5
Abused alcohol to hurt yourself	16.5	Overdosed	5.1
Not slept enough to hurt yourself	12.7*	Abused prescription medication	5.1
Made medical situations worse on purpose	15.4**	Set yourself up in a relationship to be rejected	7.0
Over-exercised to hurt yourself	24.8*	Abused laxatives to hurt yourself	1.9*

Frequency without missing cases (*N = 1, **N = 2).

In the third section, respondents assessed the occurrence and frequency of the most common:

(1) emotional triggers (*N* = 11) leading to intentional self-harm, selected from a predefined list: anger, fear, guilt, disgust, shame, hatred, injustice, feelings of failure and imperfection, sadness, unrequited love, and relationship-related emotions;
(2) emotional responses (*N* = 10) occurring after self-harm, selected from a predefined list: anger, relief, guilt, disappointment, forgiveness, shame, sadness, sense of control, sense of competence, and sense of justice.

The frequency of each emotion before and after self-harm was assessed using the same forced-choice Likert-type scale as that used for self-harm behaviors: never (0), rarely (0.25), sometimes (0.5), often (0.75), and almost always (1). The selection of emotional triggers (pre–self-harm) and emotional responses (post–self-harm) was theoretically and empirically grounded, drawing on interviews with individuals who engage in self-harm, the researchers’ professional experience, and prior empirical studies examining self-harm in relation to specific emotions: anger (Dillon et al., 2021), fear (Shi et al., 2025), guilt and shame (Sheehy et al., 2019), disgust (Smith et al., 2015), hatred (Cheng et al., 2025), injustice (Bilgin et al., 2022), feelings of failure and imperfection (Zhou et al., 2024; Gyori and Balazs, 2021), sadness (Lear et al., 2019), unrequited love (Jiang et al., 2022), relationship-related emotions (Hepp et al., 2020), relief (Carpenter et al., 2023), disappointment (Guan et al., 2024), forgiveness (Westers et al., 2012), sense of control (Bruckbauer-Schwed et al., 2025), sense of competence (Baetens et al., 2012), and sense of justice (Bilgin et al., 2022). The emotional dynamics examined in subsequent analyses (i.e., pre–post change scores) involved four emotions (*N* = 4): anger, guilt, shame, and sadness.

Within the analytical dataset, 87 individual item-level responses were missing across variables assessing self-harming behaviors and the frequency of emotions. These were missing item responses, not 87 participants with incomplete records. During the original data processing, the missing responses were coded as 0 (“never”). The questionnaire did not establish whether omission indicated absence of the phenomenon, inability to recall, unwillingness to answer, or accidental omission. In a subsequent sensitivity analysis, these 87 responses were instead treated as missing. Also, reflecting the idea of DSM-5 that self-harming behavior should be distinguished from suicidal behavior (APA, 2013), the cases reporting suicidal act were omitted. Available-case analyses were conducted for each statistical model, including only participants with complete data on the variables and participants with no suicidal behavior required for that model. The results were compared with those of the primary analysis to assess whether the handling of missing item-level responses and exclusion of suicidal behavior materially affected the main findings.

### 2.3 Statistical analysis

For Research Question 1, linear regression models were used to examine associations of gender and religious identification with the reported frequencies of individual self-harming behaviors in the full analytical sample (*N* = 158), including participants reporting no assessed self-harm. In regression analyses, religious identification was entered as an ordered predictor; no separate reference category was used, and the coefficient represents the estimated change associated with a one-category increase in religious self-identification. Research Question 2 used the subsample of participants with cumulative self-harm scores greater than zero (*N* = 130). Descriptive frequency analyses were conducted for emotions reported before and after self-harm. Differences between pre- and post–self-harm emotional states (Research Question 4) were examined using the Wilcoxon signed-rank test. To examine associations between emotions and forms of self-harm (Research Question 6), separate linear regression models were estimated for each combination of:

- emotions occurring before self-harm and specific self-harm behaviors,
- emotions occurring after self-harm and specific self-harm behaviors, and.
- changes in the frequency of occurrence of emotions (Δ anger, guilt, shame, sadness) and specific self-harm behaviors.

A second set of regression models examined associations of demographic factors (gender, religious identification) with emotional responses before and after self-harm (Research Question 3) and with the magnitude of emotional change following self-harm (Research Question 5). Research Question 7 employed linear regression models with average self-harm behavior as a covariate to determine whether the associations between demographic factors and emotional responses remained after accounting for differences in average self-harm frequency.

Given that the distributions of both self-harm behaviors and emotional variables were non-normal and significantly right-skewed, nonparametric tests and robust linear regression models were used where appropriate. Heteroskedasticity-robust (HC2) standard errors were used for linear regression models to provide inference that is robust to heteroskedasticity. Coefficients are interpreted as exploratory associations between numerically coded frequency categories rather than effects based on validated interval-level measurements. Model diagnostics, including residual-versus-fitted plots and Q-Q plots, were examined to support linearity, variance patterns, and the distribution of model residuals (not shown). Multicollinearity among predictors was assessed using variance inflation factors (VIFs); all VIF values were below 5, indicating no problematic multicollinearity. As a sensitivity analysis, ordinal logistic regression models were also fitted to the five-category ordinal anger outcomes to ensure the conclusions from the linear models were robust to treating these outcomes as ordinal rather than continuous (not shown). The significance level for all statistical tests was set at α = 0.05. *p*-values were adjusted (*p*_adj.) for multiple testing using the Benjamini-Hochberg procedure and are reported along with nominal *p* values. 95% confidence intervals were also calculated. The correction was applied within conceptually defined families corresponding to each research question and outcome set: demographic associations with self-harm behaviors, pre-self-harm emotions, post-self-harm emotions, emotion-change scores, and the three sets of emotion–behavior models. Ninety-five per cent confidence intervals were calculated. Complete model results, including non-significant associations, are provided in the Supplementary material and analytical materials available through the Figshare platform.

### 2.4 Ethical considerations

Participation in the study was voluntary and anonymous. Non-participation or withdrawal at any stage of data collection entailed no adverse consequences for participants. Prior to participation, individuals were informed about the purpose of the study and data processing procedures, and informed consent was obtained. Given the sensitive nature of the research topic, participants were offered access to psychological consultation upon study completion and were informed about available support resources. Both universities provide students with free, accredited psychological counseling services (either one-off consultations or short- to medium-term support), under the professional supervision of an independently licensed supervisor. Participants could therefore seek professional psychological assistance if completing the questionnaire caused distress or raised concerns related to self-harm. The aim of the research was reviewed and approved by the Slovak Research and Development Agency of the Ministry of Education, Science, Research and Sports of the Slovak Republic (under the project No. APVV-23-0181) and by the Ethics Committee of the Faculty of Arts, University of Ss. Cyril and Methodius in Trnava (Approval No. UCM-FF-EK 6/2023).

## 3 Results

### 3.1 Research question 1

Gender was not significantly associated with the frequency of self-harming behavior. In contrast, religious identification was negatively associated with the frequency of self-harming behavior (*p* < 0.001, *p*_adj. < 0.001), indicating that stronger religious identification was associated with lower levels of self-harming behavior. This association remained unchanged in the sensitivity analysis excluding cases with missing data and cases involving reported suicidal behavior, with religious identification remaining negatively associated with the frequency of self-harming behavior (*p* < 0.001, *p*_adj. < 0.001).

### 3.2 Research question 2

Among the most frequently reported emotions occurring prior to self-harming behavior, respondents indicated feelings of anger (*N* = 89; 68.46%), failure (*N* = 86; 66.15%), sadness (*N* = 80; 61.54%), guilt (*N* = 74; 56.92%), relationship-related emotions (*N* = 56; 43.08%), shame (*N* = 53; 40.77%), disgust (*N* = 52; 40.0%), unrequited love (*N* = 49; 37.69%), fear (*N* = 48; 36.92%), and hatred (*N* = 47; 36.15%). The least frequently reported emotional trigger for self-harm was a sense of injustice (*N* = 42; 32.31%).

According to respondents, the emotions most commonly experienced following self-harm were sadness (*N* = 76; 58.46%), relief (*N* = 66; 50.77%), disappointment (*N* = 63; 48.46%), guilt (*N* = 59; 45.38%), shame (*N* = 55; 42.31%), and a sense of control (*N* = 41; 31.53%). In contrast, the least frequently reported post–self-harm emotions were a sense of justice (*N* = 16; 12.31%), a sense of competence (*N* = 20; 15.38%), forgiveness (*N* = 26; 20.0%), and anger (*N* = 37; 28.46%).

### 3.3 Research question 3

When examining the association of demographic factors (religious identification, gender) with the occurrence of emotions before and after self-harm, religious identification was found to be negatively correlated with several emotional states experienced both before and after self-harm. Statistically significant associations are summarized in Table 2. Among the emotions experienced before self-harm, anger (*t* = −2.7498, *p* = 0.0071, *p*_adj. = 0.0467) and hatred (*t* = −2.6880, *p* = 0.0085, *p*_adj. = 0.0467) remained significantly associated with religious identification after the sensitivity analysis excluding cases with missing data and cases with reported suicidal behavior. Among the emotions experienced following self-harm, associations with religious identification remained for relief (*t* = −2.6974, *p* = 0.0083, *p*_adj. = 0.0734) and shame (*t* = −2.4858, *p* = 0.0147, *p*_adj. = 0.0734) only on nominal level.

**Table 2:** Associations between religious identification and the reported frequency of the occurrence of emotions before and after self-harm.

Emotions	*coef.*	*t*	*SE*	*95% CI*	*p*	*p_adj.*
Before self-harm						
Anger	−0.1079	−3.2311	0.0334	−0.1740 to −0.0418	0.0016**	0.0086**
Disgust	−0.0864	−2.4747	0.0331	−0.1518 to −0.0209	0.0101*	0.0269*
Shame	−0.0737	−2.4747	0.0298	−0.1327 to −0.0148	0.0147*	0.0269*
Hatred	−0.1137	−3.7515	0.0303	−0.1737 to −0.0537	0.0003***	0.0029**
Sense of Failure	−0.1016	−2.6901	0.0378	−0.1764 to −0.0269	0.0081**	0.0269*
Sadness	−0.0680	−2.0278	0.0335	−0.1343 to −0.0016	0.0447*	0.0702
Relationship-related emotions	−0.0856	−2.5235	0.0339	−0.1528 to −0.0185	0.0129*	0.0269*
After self-harm						
Relief	−0.1544	−4.6202	0.0334	−0.2205 to −0.0883	<0.001***	<0.001***
Shame	−0.0844	−2.8727	0.0294	−0.1426 to −0.0263	0.0048**	0.0239*
Sense of Control	−0.0849	−2.7009	0.0314	−0.1471 to −0.0227	0.0079**	0.0262*
Sense of Competence	−0.0450	−2.0322	0.0221	−0.0888 to −0.0013	0.0442*	0.1095

*p ≤ 0.05; **p ≤ 0.01; ***p ≤ 0.001.

No statistically significant associations between gender and the occurrence of emotions before or after self-harm were observed.

### 3.4 Research question 4

Research Question 4 examined which emotions (anger, guilt, shame, and sadness) showed a significant change following self-harm compared to their levels prior to self-harm. The change score was calculated as the value after self-harm minus the value before self-harm, and negative values indicate a decrease in the reported frequency of the respective emotion following self-harm. The paired analysis identified statistically significant changes in three emotions. Although the frequency of occurrence of all four emotions decreased after self-harm, only the decreases in anger, sadness, and guilt reached statistical significance (Table 3). Two of the three changes in the reported levels of emotions remained statistically significant after the sensitivity analysis—guilt (*t* = 293, *p* = 0.0401) and anger (*t* = 62, *p* < 0.001), both of which showed a decline following self-harm.

**Table 3:** Changes in the frequency of occurrence of emotions before and after self-harm (paired nonparametric Wilcoxon signed-rank test).

Emotion	Change score	Statistic	*p*
Anger	−0.25	168	<0.001***
Guilt	−0.0577	752.5	0.0309*
Shame	−0.0077	929	0.7334
Sadness	−0.0673	620	0.0057**

*p ≤ 0.05; **p ≤ 0.01; ***p ≤ 0.001.

### 3.5 Research question 5

Research Question 5 examined whether gender and religious identification were associated with the magnitude of change in emotional experience (anger, guilt, shame, and sadness) before and after self-harm. Gender showed no statistically significant association with emotional change. Religious identification was associated only with anger, with a post–self-harm decrease in anger in connection with more intense religious orientation (*t* = 2.4110, *p* = 0.01733). However, this association did not remain significant after correction (*p_*adj. = 0.1814). These results were retained also after the sensitivity analysis.

### 3.6 Research question 6

The first part of Research Question 6 focused on analyzing the relationship between the occurrence of emotions prior to self-harm and subsequent forms of self-harming behavior. Linear regression models examining emotional states prior to self-harm and the subsequent occurrence of specific forms of self-harm revealed several connections (see Table 4). Statistically significant associations (after Benjamini–Hochberg correction) were identified between anger and hitting, disgust and starving, and between hatred and torturing with self-defeating thoughts. These associations remained significant also after sensitivity analysis.

**Table 4:** Associations between selected emotions prior to self-harm and specific forms of self-harming behavior.

Emotion prior to self-harm	Form of self-harming behavior	*coeff.*	*t*	*SE*	*95% CI*	*p*	*p_adj.*
Anger	Hit yourself	0.3682	4.3852	0.0840	0.2019 to 0.5345	<0.001***	<0.001***
	Scratched yourself on purpose	0.2252	2.2209	0.1014	0.0244 to 0.4259	0.0283*	0.1885
	Attempted suicide	−0.1269	−2.4567	0.0516	−0.2291 to −0.0246	0.0155*	0.1548
Fear	Attempted suicide	−0.1134	−2.1715	0.0522	−0.2169 to −0.0010	0.0319*	0.6359
Guilt	Cut yourself on purpose	0.2117	1.9819	0.1068	0.0002 to 0.4233	0.0498*	0.2491
	Abused alcohol to hurt yourself	0.2433	2.2077	0.1102	0.0251 to 0.4616	0.0292*	0.2102
	Made medical situations worse on purpose	−0.2353	−2.5497	0.0923	−0.4181 to −0.0526	0.0121*	0.2102
	Attempted suicide	0.2028	2.1762	0.0932	0.0183 to 0.3873	0.0315*	0.2102
Disgust	Abused alcohol to hurt yourself	0.2216	2.0806	0.1065	0.0107 to 0.4326	0.0396*	0.3964
	Starved yourself to hurt yourself	0.3772	3.8385	0.0983	0.1826 to 0.5717	<0.001***	0.0040**
Shame	Made medical situations worse	0.1885	2.2456	0.0839	0.0223 to 0.3548	0.0266*	0.5045
Hatred	Cut yourself on purpose	0.2306	2.2494	0.1025	0.0276 to 0.4337	0.0263*	0.0954
	Overdosed	0.1264	2.2160	0.0570	0.0134 to 0.2393	0.0286*	0.0954
	Made medical situations worse	0.2075	2.0494	0.1013	0.0070 to 0.4081	0.0426*	0.1218
	Engaged in emotionally abusive relationships	0.2944	2.4436	0.1205	0.0558 to 0.5330	0.0160*	0.0954
	Attempted suicide	0.1478	2.2654	0.0652	0.0186 to 0.2770	0.0253*	0.0954
	Exercised an injury on purpose	0.2365	2.6219	0.0902	0.0579 to 0.4150	0.0099**	0.0954
	Tortured yourself with self-defeating thoughts	0.3892	3.3066	0.1177	0.1561 to 0.6223	0.0013**	0.0250*
Relationship-related emotions	Cut yourself on purpose	0.2444	2.3624	0.1034	0.0395 to 0.4492	0.0198*	0.1979
	Over-exercised to hurt yourself	0.3343	2.6988	0.1239	0.0890 to 0.5796	0.0080**	0.1596

*p ≤ 0.05; **p ≤ 0.01; ***p ≤ 0.001.

The second part of Research Question 6 examined the relationship between emotions reported after self-harm and specific forms of self-harming behavior. Linear regression analyses identified several connections between forms of self-harm and subsequent emotional experiences however, none remained statistically significant after adjustment using the Benjamini–Hochberg procedure, with one exception: self-harm involving the misuse of laxatives remained significantly associated with guilt after correction (see Table 5). However, after employing the sensitivity analysis, this result did not reach the level of statistical significance. In contrast, after excluding participants with missing data and those who reported suicide attempts, “Cut yourself on purpose” emerged as the form of self-harming behavior nominally associated with anger (*t* = −2.4989, *p* = 0.0145), relief (*t* = 2.1680, *p* = 0.0332), and sense of justice (*t* = −2.7621, *p* = 0.0071).

**Table 5:** Associations between specific forms of self-harming behavior and subsequent emotional experiences.

Form of self-harming behavior	Emotion after self-harm	*coef.*	*t*	*SE*	*95% CI*	*p*	*p_adj.*
Cut yourself on purpose	Anger	−0.1765	−2.0415	0.0864	−0.3478 to −0.0051	0.0436*	0.1636
	Relief	0.3462	2.4781	0.1397	0.0693 to 0.6232	0.0249*	0.1474
	Sense of Justice	−0.1410	−1.9902	0.0708	−0.2814 to −0.0006	0.0491*	0.1636
Banged your head on purpose	Relief	0.3479	2.2746	0.1530	0.0448 to 0.6511	0.0249*	0.1226
	Guilt	0.4105	2.1140	0.1942	0.0256 to 0.7954	0.0368*	0.1226
	Disappointment	0.5257	2.3488	0.2238	0.0821 to 0.9693	0.0206*	0.1226
	Sense of Control	0.3732	1.9845	0.1880	0.0005 to 0.7458	0.0423*	0.1387
Attempted suicide	Sense of Control	0.4949	2.1747	0.2275	0.0439 to 0.9459	0.0439*	0.1591
	Sense of Competence	0.4237	2.5114	0.1687	0.0893 to 0.7581	0.0113*	0.1127
Abused laxatives to hurt yourself	Guilt	1.1052	3.0546	0.3618	0.3881 to 1.8224	0.0028**	0.0283*

*p ≤ 0.05; **p ≤ 0.01.

When examining the relationship between the magnitude of change in emotional experience (anger, guilt, shame, and sadness) and the form of self-harm, linear regression analyses identified several forms of self-harming behavior that were associated with changes in emotional experience. Statistically significant associations (after Benjamini–Hochberg correction) remained in behaviors “Hit yourself” and “Made medical situations worse on purpose” with changes in emotional experience of anger (Table 6). Analysis including only participants without missing data and without suicidal intents expanded this list to include also negative associations with “Banged your head on purpose” (*t* = −1.9969, *p* = 0.0493, *p*_adj. = 0.1971) and “Over-exercised to hurt yourself” (*t* = −2.0034, *p* = 0.0486, *p*_adj. = 0.1943), however only on the level of nominal significance.

**Table 6:** Forms of self-harm with associations with the changes in emotional experience.

Emotion	Form of self-harming behavior	*coef.*	*t*	*SE*	*95% CI*	*p*	*p_adj.*
Anger	Hit yourself	−0.4239	−3.0370	0.1396	−0.7006 to −0.1473	0.0030*	0.0120*
	Overdosed	−1.0740	−2.0613	0.5210	−2.1067 to −0.0413	0.0417*	0.1409
	Made medical situations worse on purpose	−0.6023	−3.5947	0.1676	−0.9344 to −0.2702	<0.001***	0.0020**
	Banged your head on purpose	−0.3983	−2.3981	0.1661	−0.7275 to −0.0691	0.0182*	0.0727
	Over-exercised to hurt yourself	−0.3688	−2.3623	0.1561	−0.6782 to −0.0594	0.0199*	0.07973
Sadness	Prevented wounds from healing	0.3702	2.1249	0.1742	0.0249 to 0.7154	0.0359*	0.1434

*p ≤ 0.05; **p ≤ 0.01; ***p ≤ 0.001.

### 3.7 Research question 7

Self-harming behavior was significantly negatively (*t* = −4.5277, *p* < 0.001, *p*_adj. < 0.001) associated with religious identification. After accounting for the differences in the levels of self-harming behavior and gender, no significant associations were found between religious identification and either the frequency of emotions following self-harm or changes in emotional states. Among the emotions assessed prior to self-harm, only fear showed a nominal association with religious identification (*t* = 2.4304, *p* = 0.0165, *p*_adj. = 0.1814). These results were retained after the sensitivity analysis.

## 4 Discussion

A general finding emerging from the analyses is that gender did not function as a significant factor in the relationships between emotions and self-harm. Although it is well established that the prevalence of self-harm is substantially higher among women than among men (Moloney et al., 2024), gender was not associated with (1) the occurrence of emotions prior to self-harm, (2) the occurrence of emotions following self-harm, nor with (3) the magnitude of change in emotional experience (anger, guilt, shame, sadness) following self-harm relative to the pre–self-harm state. Thus, although women appear to choose self-harm as a maladaptive strategy for coping with distress and negative emotions more frequently than men (Moloney et al., 2024), no statistically significant gender associations were detected in the present sample. The reported frequencies of emotions before and after self-harm and the calculated emotion-change scores were also not significantly associated with gender. The relatively small convenience sample and substantial gender imbalance (110 women and 20 men) limit the generalizability of the findings and may have reduced the statistical power to detect gender-related associations. No *a priori* power analysis was conducted. Accordingly, the absence of statistically significant gender associations should not be interpreted as evidence of gender equivalence or as demonstrating the absence of gender-related differences. These findings should be considered inconclusive and require replication in larger, more gender-balanced samples.

### 4.1 Most common emotions before and after self-harm

A majority of participants (more than 50%) reported that the most common emotional triggers leading to self-harm were anger (*N* = 89; 68.46%), feelings of failure (*N* = 86; 66.15%), and sadness (*N* = 80; 61.54%). These are negatively valenced emotions experienced as aversive, for which individuals naturally seek strategies to eliminate or at least reduce their intensity. Multiple studies indicate that individuals who self-harm often exhibit deficits in emotion regulation (Wolff et al., 2019; Clapham and Brausch, 2022), which substantially limits their capacity to manage these emotional states. Limited access to emotion regulation strategies (Brausch et al., 2022) may therefore contribute to the selection of self-harm as a maladaptive strategy for managing aversive emotions. However, the present retrospective data establish neither temporal causality nor the subjective intensity of these emotions; they measure only their reported frequency of occurrence in relation to self-harm.

Anger is distinctive insofar as individuals may frequently direct it toward the self, such that self-harm serves not only as a means of coping with intense emotions but also as a way to discharge anger through self-injury—that is, as self-directed aggression. This interpretation is supported by other findings. For example, Dierickx et al. (2026), in a sample of young adults, found that adults with a history of self-harm reported higher anger than peers without such history, specifically higher-than-usual anger toward the self but not toward others. Moreover, Dillon et al. (2021) identified anger as a significant predictor of self-harm. Feelings of failure may emerge directly from difficulties in regulating and processing emotions; this experience further accumulates existing distress and increases the likelihood of responses aimed at relieving perceived tension. As Wang (2021) notes, failures of emotion regulation can stimulate the desire to engage in stress-reduction behaviors, including self-harming behavior. Sadness commonly accompanies multiple negatively valenced emotional states. Sadness has been found to be a positive predictor of urges to engage in self-harm (Bresin et al., 2013). Dillon et al. (2022) reported that both anger and sadness predicted self-harm, with the distinction that anger was more directly linked to self-harming behavior, whereas sadness primarily increased urges toward self-harm.

Regarding emotions following self-harm, the most frequent emotional responses were sadness (*N* = 76; 58.46%), relief (*N* = 66; 50.77%), disappointment (*N* = 63; 48.46%), guilt (*N* = 59; 45.38%), and shame (*N* = 55; 42.31%). The prevalence distribution indicates greater variability in emotional experience following self-harm than prior to self-harm: unlike the pre–self-harm emotions, only relief exceeded the 50% threshold, while the remaining post-self-harm emotions were reported by fewer than half of the respondents. It may therefore be inferred that the emotional states preceding self-harm were more uniformly reported than the emotional responses following self-harm.

Notably, among the five most frequently experienced post–self-harm emotions, four (sadness, disappointment, guilt, shame) are negatively valenced. An important finding, therefore, is that although self-harm is commonly conceptualized as a means of alleviating emotional distress (Richmond et al., 2017; Zhou et al., 2025), individuals predominantly experienced negative emotions after engaging in self-harm. This pattern underscores the maladaptive nature of self-harm as a coping strategy and the findings are consistent with the cyclical hypothesis of self-harm (Sutton, 2007), which emphasizes the re-emergence of negative emotions following self-harm. The presence of negative emotions following self-harm was also demonstrated in the study by Chapman and Dixon-Gordon (2007), in which the authors reported the occurrence of anger (6.45%), tension (6.45%), guilt (9.68%), and sadness (12.90%). Thus, the relief reported as the only positively valenced post–self-harm emotion appears temporary and partial, attenuated by the concurrent experience of multiple negative emotional states. This, in turn, may lead to renewed tension and the accumulation of unprocessed emotions, thereby increasing the likelihood of subsequent self-harm.

### 4.2 Associations of religious identification with emotions before and after self-harm

Of the two demographic factors examined (gender and religious identification), only religious identification was associated with the studied variables. After Benjamini–Hochberg adjustment, religious identification was significantly associated with six of the eleven emotional triggers (emotions prior to self-harm) and three of the ten emotional responses (emotions following self-harm). In all cases, the frequency of occurrence of both emotional triggers and emotional responses was negatively associated with religious identification. This applied to negatively valenced emotions (anger, guilt, disgust, shame, etc.) as well as positively valenced states (relief, forgiveness, sense of control, etc.). After the sensitivity analysis, significant associations with religious identification remained for anger and hatred prior to self-harm and for relief and shame following self-harm; all associations were statistically significant and negative.

The reason why religious identification is negatively associated with emotions experienced before and following self-harm may lie in religious contexts. Although denominational affiliation was not assessed, the predominance of Christianity in the sociocultural context may warrant considering whether religious identification is associated with moderation and self-control in emotional experience and expression. Temperance, understood as a virtue emphasizing moderation, self-control, and balance, is promoted not only in Christianity but also in other religious traditions, including Islam. However, it remains unclear whether such patterns would reflect differences in the cognitive appraisal and reporting of emotions (i.e., outward moderation) or genuine differences in the frequency of experienced emotional distress. This raises the possibility that religiosity could be associated with lower levels of self-harm, although the mechanisms underlying such an association remain uncertain.

Individuals with higher levels of religious identification showed significantly lower engagement in self-harming behavior (*p* < 0.001, *p*_adj. < 0.001) however, it is unclear whether religious identification itself is associated with lower emotionality before and after self-harm, or whether it is the frequency of self-harm itself reducing the emotionality. In Research Question 7, we examined whether the average frequency of self-harm, gender or religious identification are significantly associated with emotions before and after self-harm. As expected, for most of the emotions both prior and following the act, average self-harm frequency served best to explain the emotionality. However, religion was significantly associated with fear before self-harm even after average frequency of self-harm was treated as a separate independent variable (*t* = 2.4304, *p* = 0.0165, *p*_adj. = 0.1814). A possible explanation may once again lie in the values upheld by the majority of religious denominations present within the cultural and social context of the research sample. Prior to engaging in self-harm, individuals may recognize that such behavior is inconsistent with established norms, as the intentional infliction of bodily harm is considered wrongful conduct in most religious traditions. In addition to feelings of fear arising from the violation of these norms, fear of self-harm among religious individuals may also be intensified by concerns about the punishment believed to follow wrongful conduct (sin) in many religious traditions. However, these interpretations remain hypothetical and would require further investigation to establish their validity.

Importantly, the present study did not assess denominational affiliation, specific religious beliefs concerning self-harm, religious coping, moral conflict, perceived religious prohibitions, or temperance and self-control as psychological constructs. Religious identification was operationalized solely through broad self-reported identification. Therefore, the observed negative associations between religious identification and self-harm cannot be attributed to any specific religious, psychological, or moral mechanism, nor can they be interpreted as evidence of a causal or protective effect of religious identification. The mechanisms proposed above—including moderation, self-control, religious coping, and moral or denominational influences—should consequently be regarded as tentative, theoretically plausible hypotheses requiring independent empirical testing with validated multidimensional measures of religiosity and designs capable of examining these mechanisms directly. Research examining religiosity and self-harm remains relatively limited; however, a recent meta-analysis reported a small but statistically significant negative correlation between religious identification and self-harm (Haney, 2020). The potential role of religiosity in self-harm and its correlates, including emotional experiences, therefore warrants further investigation.

### 4.3 Changes in emotional experience, the role of religious identification, and self-harm form-specific associations

When examining changes in the frequency of selected emotions (anger, guilt, shame, sadness) before versus after self-harm, statistically significant differences were identified for anger, guilt, and sadness; after sensitivity analysis, results remained significant for anger and guilt. The most important finding is that the reported frequency of occurrence of emotions slightly decreased but still remained present. This result again highlights the maladaptive character of self-harm, which appears to be an inappropriate strategy for reducing psychological distress in many respects. Our findings indicate that, after engaging in self-harm, the examined negative emotions were reported less frequently. These results are consistent with qualitative studies focusing on individuals’ reflections after self-harm (see, e.g., Limsuwan et al., 2023), which describe perceptions of uselessness, unhelpfulness, and unwanted consequences of self-harm and a subsequent recognition of the adverse consequences of this coping strategy. They are also indirectly compatible with Sutton’s cyclical hypothesis of self-harm, which describes an increase in shame, guilt, self-disgust, and self-hatred in the final phases of the cycle (Sutton, 2007).

In the context of emotional change, gender showed no statistically significant association with emotional change whilst religious identification was associated only with change in levels of anger with the tendency toward decreased negative emotional frequency following self-harm. However, this association should be treated as a tendency as it did not remain significant after the Benjamini-Hochberg procedure, nor the sensitivity analysis.

The tendency to report lower frequency of anger with the religious orientation prior to self-harm may reflect differences in emotion regulation, meaning-making, or normative evaluation of anger and self-harm in a religious context. Religious beliefs may be associated with processes such as self-control, forgiveness, acceptance, or reinterpretation of anger-provoking situations. Alternatively, the lower frequency of anger could reflect a shift of primary anger toward other self-evaluative emotions such as guilt or shame rather than a more adaptive emotional outcome. To further examine these possibilities, we also tested these parameters, in which lower levels of anger connected with religiosity identification were shown to be positively related to an overall lower frequency of self-harm. However, these findings do not establish that religious identification has a protective effect, nor can the observed associations be attributed to self-control, forgiveness, religious coping, moral conflict, fear of punishment, or any other specific mechanism. Furthermore, the association between religious identification and anger warrants cautious interpretation for two reasons. First, it did not remain statistically significant following corrections for multiple comparisons. Second, religious identification was assessed using a broad ordinal self-identification variable rather than a validated, multidimensional measure of religiosity. Consequently, the observed association should not be interpreted as evidence of a specific religious or psychological mechanism underlying anger or self-harm.

When examining changes in the frequency of selected emotions (anger, guilt, shame, and sadness) before versus after self-harm and their associations with specific forms of self-harming behavior, associations were identified for changes in anger and sadness. However, after correction for multiple comparisons, only the associations between changes in anger and the self-harm forms “Hit yourself” and “Made medical situations worse on purpose” remained statistically significant. Most notably, change in anger was negatively associated with both forms of self-harm, indicating that higher frequencies of these self-harming behaviors were associated with smaller changes in anger. The analysis of the associations between changes in the frequency of selected emotions and specific forms of self-harming behavior, conducted on a sample excluding cases with missing data and cases involving suicidal behavior, expanded the list of forms of self-harming behavior to include two additional forms, “Banged your head on purpose” and “Over-exercised to hurt yourself,” both of which were significantly associated with changes in anger. In both cases, the association was again negative. These findings are consistent with the potentially maladaptive character of self-harm, which may function as a short-term strategy for reducing negative emotional states but may become less effective over time. However, this interpretation remains tentative and requires further examination using methods better suited to assessing temporal and causal processes, which may provide an important direction for future research.

### 4.4 Emotional triggers and responses associated with specific forms of self-harm

At the nominal significance level, hatred preceding self-harm showed the biggest number of associations with several forms of self-harm; however, most of these associations did not remain statistically significant after correction for multiple testing. The only significant association was identified between hatred and torturing with self-defeating thoughts. Hatred is a highly negatively valenced emotion. Empirical conceptual analyses of hate and related constructs (dislike, anger, contempt, and disgust) indicate that participants feel consistently more emotionally aroused, threatened, and inclined toward attack-oriented behaviors when experiencing hate compared with other moral emotions (Martínez et al., 2022). When the target of hate is the self, this corresponds to self-hatred. Self-hatred has been identified as a significant factor increasing the likelihood of self-harming behavior (Nagy et al., 2021; Nilsson et al., 2022). Injustice, sadness, and unrequited love did not show significant association with any of the monitored forms of self-harm. Injustice and unrequited love (and in some cases sadness) reflect difficulties primarily in the interpersonal domain. It appears that, in our sample, these experiences may not constitute as strong a source of psychological distress as intrapersonal difficulties. Most emotions that serve as triggers for specific forms of self-harm (anger, fear, guilt, disgust, shame, hatred) reflect an individual’s internal struggle with personal difficulties. Accordingly, future research on emotional predictors of self-harm may benefit from prioritizing intrapersonally oriented emotional triggers.

Also for other preceding emotions, statistically significant associations with specific forms of self-harm were observed. Notably, anger was associated with “hit yourself,” whereas disgust was associated with “starved yourself to hurt yourself.” Self-harm is often characterized as highly compulsive behavior with limited perceived control (Carenys and Adan, 2025). In the context of a rapid escalation of anger, hitting oneself may represent a particularly immediate form of self-harm, as it does not require any additional tools or objects, such as sharp objects used for cutting or a heat source used for burning. The association between disgust and restricting food intake may, hypothetically, be understood in relation to the nature of the emotion itself. Disgust is considered, among other functions, a disease-avoidance emotion, with its primary biological function being to protect the organism against illness, disease, and contamination (Davey, 2011). Thus, the selection of a self-harming behavior involving food restriction may conceptually correspond to this underlying mechanism. Moreover, nausea is a prominent somatic manifestation of disgust (Alladin et al., 2024) and may contribute to an aversive response toward food. Taken together, these findings suggest that specific emotions experienced prior to self-harm may be significantly associated with the particular form of self-harming behavior selected by an individual.

Analyses of post–self-harm emotional responses as a function of the enacted form of self-harm indicated that the form of self-harm associated with the largest number of post–self-harm emotional responses was “Banged your head on purpose.” It was positively associated with reported relief, sense of control, disappointment, and guilt. In other words, using this method was associated with an increased occurrence of relief and a sense of control, but also increased disappointment and guilt. However, these findings should be interpreted with caution, as they did not remain statistically significant after adjustment for multiple testing using the Benjamini–Hochberg procedure.

Sensitivity analysis identified associations between cutting and anger (negative association), relief (positive association) and sense of justice (negative association). Ambivalence in post–self-harm affect thus appears to be a characteristic feature of this experience. Reports by individuals who self-harm indicate that, rather than eliminating tension, distress, and psychological difficulties as expected, relief is temporary; moreover, negative psychological states do not disappear completely and may re-emerge relatively soon after the self-harm act, accompanied by another wave of negative emotional reactions: “…I feel like hurting myself doesn’t help much in the end.” and “…I used to think the more I hurt myself, the better I would feel. But in fact, it only made me worse” (Limsuwan et al., 2023, p. 6).

The only emotion following self-harm that was statistically significantly associated with a specific form of self-harm (“abused laxatives to hurt yourself”) was guilt. One possible explanation may relate to the self-directed and potentially self-punitive nature of this specific behavior. Unlike some more immediately observable forms of self-harm, such as cutting or hitting oneself, laxative misuse may involve a less visible form of bodily harm. Following the behavior, individuals may experience guilt as a self-conscious emotional response to having intentionally harmed themselves or engaged in behavior that they subsequently perceive as unnecessary, harmful, or difficult to control. Importantly, however, the available evidence does not establish that laxative misuse causes guilt, nor can the present cross-sectional findings determine the direction of this association. Rather, the elevated occurrence of guilt associated with this particular form of self-harm may indicate that the behavior is connected with increased self-evaluative distress. This possibility warrants further investigation, particularly in studies examining the temporal dynamics of specific self-harming behaviors and subsequent emotional responses.

## 5 Limitations and future directions

Several limitations of the present study should be considered. Data were collected from a relatively homogeneous sample (psychology and law students, i.e., humanities disciplines). Such participants are trained to reflect on behavioral motivation, which may have facilitated more accurate reporting; however, this also limits the broad generalizability of the findings. Extending the research to a more heterogeneous sample (not only in education but also across other demographic and sociocultural parameters), as well as increasing the sample size, would improve precision and support broader applicability.

Because the study centered on the experience and reflection of emotions, it relied on self-report measures, which depend on respondents’ willingness to answer truthfully and their capacity to accurately reflect on and report their own psychological states. This imposes important limitations. Participants provided retrospective self-report data collected at a single time point, covering their lifetime experiences, and generalized the frequency of emotions associated with the periods before and after self-harming behavior. Thus, the reported experiences could not be independently verified, nor were they obtained through repeated assessments conducted immediately before and after specific episodes of self-harm. Validation via convergent measures was not feasible in this context, as systematic research and established methodologies in this specific area remain limited. Although the authors compiled lists of emotional triggers and emotional responses based on the literature, examining additional emotions would likely be beneficial. In particular, the assessment of differences in emotional intensity before and after self-harm appears especially promising; future work should analyze a substantially broader list of emotions. However, the present study was not designed to assess changes in the intensity of emotional experiences before and after self-harm, nor to capture their precise temporal dynamics (e.g., the latency to their re-emergence following a self-harming episode). Rather, our analysis was restricted to the reported recurrence or absence of particular emotions in relation to self-harming behavior.

A similar limitation applies to the religious identification variable. Participants were broadly categorized as believers, non-believers, or uncertain, with the “believer” category encompassing individuals identifying with any religious, denominational, or metaphysical framework. Accordingly, this variable may be more accurately characterized as self-reported worldview or religious identification rather than religiosity per se. It does not capture the depth, intensity, or specific nature of participants’ beliefs, but merely reflects how they self-identified in the questionnaire. Importantly, the category “believer” should not be interpreted as representing a homogeneous level or form of religiosity. In the present study, this category was based solely on participants’ self-identification and broadly encompassed all respondents who identified themselves as believers, irrespective of the specific nature, intensity, or behavioral expression of their beliefs. It may therefore include individuals characterized by deeply internalized (intrinsic) religiosity, those whose religious orientation is predominantly extrinsic, as well as individuals whose identification as believers is primarily formal and may not be accompanied by substantial internal commitment or observable religious practice. Consequently, membership in the “believer” category should be understood as a broad self-reported religious or worldview identification rather than as evidence of a specific type or degree of religiosity. This heterogeneity substantially limits the interpretation of the observed association between the “believer” category and self-harm. In particular, the present findings do not provide sufficient evidence to conclude that religious identification per se constitutes a protective factor against self-harm (Good et al., 2011). Such an interpretation requires considerable caution, especially given that the response “I do not know,” treated as a neutral category in our operationalization, may not necessarily indicate the absence of a religious orientation and could, in some cases, be compatible with a quest orientation toward religion (Batson et al., 1993). A more rigorous examination of the relationship between religiosity and self-harm would therefore require standardized and validated measures capable of differentiating the intensity, dimensions, and orientations of religiosity.

Another limitation is that the modified version of the Self-Harm Inventory used in this study has not yet undergone formal psychometric validation. The modifications were intended to improve the instrument’s suitability for Slovak participants, and previous research has demonstrated good internal consistency for the modified version. However, internal consistency alone does not establish the construct validity of this heterogeneous modified measure. The validity and reliability of the modified frequency scoring have not yet been formally established—further psychometric research is therefore needed to validate the scoring approach and the list of self-harming behaviors.

We also acknowledged that the distribution of the data is right-skewed. To ensure the robustness of the approach, heteroskedasticity-robust standard errors were used to reduce the sensitivity of statistical inference to heteroskedasticity. Additionally, diagnostic assessment of residual behavior did not indicate substantial violations of the model assumptions. Multicollinearity was also evaluated using variance inflation factors (VIFs), with no substantial collinearity observed among the predictors. However, the bounded ordinal coding, right-skewed distributions, and numerous zero values limit the fit between the data and linear-model assumptions. HC2 standard errors provide heteroskedasticity-robust inference but do not make ordinal frequency categories equivalent to continuous interval-level measurements. Model diagnostics and VIFs did not indicate major additional problems, and an ordinal-logistic sensitivity analysis for anger produced substantively similar conclusions. Because this sensitivity analysis was limited to anger, the remaining linear-model findings should be interpreted as exploratory.

A limitation of this study concerns also the treatment of missing item-level data. During the initial data preparation, missing values were assigned a score of 0, corresponding to “never.” However, the questionnaire did not capture the reasons for non-response, making it impossible to determine whether participants intended to indicate the absence of behavior or emotion, were unable to recall the relevant information, chose not to respond, or omitted the item unintentionally. As the underlying causes of missingness could not be established retrospectively, there was insufficient evidence to support a reliable alternative recoding strategy. Nevertheless, this approach may have introduced systematic bias, as treating missing responses as an absence of the measured phenomenon could have led to an underestimation of reported frequencies and influenced the observed associations between variables. Future questionnaires should distinguish “never,” “cannot recall,” “prefer not to answer”, and accidental omission and should prespecify an appropriate missing-data strategy.

A further limitation concerns the content validity of the assessed emotional triggers and responses. Despite ongoing efforts to refine the terminology used to describe emotional states, individuals differ in how they understand emotion terms based on unique experiences, as well as cultural context, upbringing, and related factors (Barrett, 2004). Emotional states are not always clearly delineated and may overlap conceptually and experientially. Feelings and emotions often constitute multidimensional continua that can be difficult to capture verbally, as they may be (and often are) mixed. Accordingly, future research would benefit from complementary approaches that allow more precise assessment of discrete emotional states and underlying intentions.

## 6 Conclusion

The present study provides further insight into the emotional experiences associated with self-harm among young adults, with particular attention to emotions preceding and following self-harming behavior. The most frequently reported emotional triggers were anger, feelings of failure, sadness, guilt, and shame, supporting the conceptualization of self-harm as a maladaptive emotion-regulation strategy in the context of pronounced intrapersonal distress. Following self-harm, negative emotions remained prominent, although anger, guilt, and sadness decreased significantly, a pattern compatible with elements of cyclical models of self-harm; however, the retrospective cross-sectional design does not permit conclusions about temporal or causal mechanisms. Gender was not significantly associated with the examined emotional variables, although the substantial gender imbalance and limited statistical power warrant cautious interpretation of this finding. Religious identification was negatively associated with self-harm frequency and with the reported frequency of several emotions, but these associations cannot be interpreted as evidence of a causal or protective effect of religiosity. Finally, selective associations between particular emotions and specific forms of self-harm suggest potentially differentiated emotional patterns that warrant further investigation in larger and more diverse samples.

## Data Availability

The raw data supporting the conclusions of this article can be found in the dataset published on the Figshare platform at: https://doi.org/10.6084/m9.figshare.31028782.
