# Supplementary material for: Self-harm in the cycle of (moral) emotions: an exploratory analysis of emotional triggers and responses
Source: Front Psychol. 2026 Oct 28;17:1783881. doi: 10.3389/fpsyg.2026.1783881 (PMC13751391; doi:10.3389/fpsyg.2026.1783881)
Supplement: Supplementary file 1 [file Data_Sheet_1.PDF]

```
rm(list=ls())

library(tidyverse)
library(broom)
library(factoextra)
library(estimatr)
library(car)
library(ordinal)

# Load data
data <- read.csv("Sebapoškodzovanie a morálne emócie(1-165).csv")

# --- Clean data ---

data <- data %>%
  mutate(across(where(is.numeric), ~replace_na(., 0)))

# --- Extract variable groups ---
demographics <- data %>% select(ID, starts_with("Sex"), starts_with("Religion"))
behaviors <- data %>% select(ends_with("_beh"))
emotions_before <- data %>% select(ends_with("_emob"))
emotions_after <- data %>% select(ends_with("_emoa"))

# --- Combine into one dataset ---
data_clean <- bind_cols(demographics, behaviors, emotions_before, emotions_after)

# --- SH only dataset ---
data_sh_only <- data_clean %>%
  mutate(total_behavior = rowSums(across(ends_with("_beh")), na.rm = TRUE)) %>%
  filter(total_behavior > 0)

demographics_sh_only <- data_sh_only %>% select(ID, starts_with("Sex"), starts_with("Religion"))
behaviors_sh_only <- data_sh_only %>% select(ends_with("_beh"))
emotions_before_sh_only <- data_sh_only %>% select(ends_with("_emob"))
emotions_after_sh_only <- data_sh_only %>% select(ends_with("_emoa"))

# --- Emotion deltas ---
common_emotions <- intersect(
  str_remove(names(emotions_before_sh_only), "_emob$"),
  str_remove(names(emotions_after_sh_only), "_emoa$")
)

emotions_before_common <- emotions_before_sh_only %>% select(ends_with(paste0(common_emotions,
"_emob")))
emotions_after_common <- emotions_after_sh_only %>% select(ends_with(paste0(common_emotions,
"_emoa")))

delta_emotions <- emotions_after_common - emotions_before_common
colnames(delta_emotions) <- paste0("delta_", common_emotions)
delta_df <- bind_cols(data_sh_only, delta_emotions)

# ===============================
# PAIRED TESTS: BEFORE vs AFTER (NO BEHAVIOR)
# ===============================

paired_overall <- map_df(common_emotions, function(emotion) {

  before <- emotions_before_sh_only[[paste0(emotion, "_emob")]]
  after  <- emotions_after_sh_only[[paste0(emotion, "_emoa")]]

  test <- wilcox.test(after, before, paired = TRUE)

  tibble(
    emotion = emotion,
```

```
    mean_before = mean(before, na.rm = TRUE),
    mean_after  = mean(after, na.rm = TRUE),
    mean_delta  = mean(after - before, na.rm = TRUE),
    t_value     = unname(test$statistic),
    p_value     = test$p.value
  )
})

write.csv(
  paired_overall,
  "plots/Paired_Before_After_Overall.csv",
  row.names = FALSE
)

# ================================
# LINEAR MODELS
# ================================

# --- Plot function ---
plot_coeffs <- function(df, title, filename) {
  p <- df %>%
    filter(term != "(Intercept)") %>%
    mutate(signif = p.value < 0.05) %>%
    ggplot(aes(x = term, y = estimate, color = signif)) +
    geom_point() +
    geom_errorbar(aes(ymin = estimate - std.error, ymax = estimate + std.error), width = 0.2) +
    facet_wrap(~Outcome, scales = "free_y") +
    theme_minimal(base_size = 10) +
    coord_flip() +
    labs(title = title, y = "Coefficient", x = "Predictor") +
    scale_color_manual(values = c("grey50", "red")) +
    theme(strip.text = element_text(size = 8))

  ggsave(filename = file.path("plots", filename), plot = p, width = 12, height = 8)
}

# --------------------------------
# Helper function: robust models + FDR + optional VIF
# --------------------------------
fit_robust_models <- function(outcome_vars, predictors, data, check_vif = FALSE, set_name = NULL)
{

  results <- map_df(outcome_vars, function(var) {

    formula <- as.formula(paste(var, "~", predictors))

    lm_robust(formula, data = data) %>%
      tidy() %>%
      mutate(Outcome = var)
  })

  # FDR correction
  results <- results %>%
    group_by(term) %>%
    mutate(
      p_adj = p.adjust(p.value, method = "fdr"),
      nominally_significant = p.value < 0.05,
      fdr_significant = p_adj < 0.05
    ) %>%
    ungroup()

  # VIF + simple diagnostic plots
  if (check_vif & !is.null(set_name)) {

    # VIF
```

```
    vif_model <- lm(
      as.formula(paste(outcome_vars[1], "~", predictors)),
      data = data
    )

    vif_table <- tibble(
      Predictor = names(vif(vif_model)),
      VIF = as.numeric(vif(vif_model))
    )

    write_csv(
      vif_table,
      paste0("VIF_", set_name, ".csv")
    )

    # Diagnostic plots
    png(
      paste0("Diagnostics_", set_name, ".png"),
      width = 1200,
      height = 600
    )

    par(mfrow = c(1, 2))

    plot(vif_model, which = 1)  # Residuals vs fitted
    plot(vif_model, which = 2)  # Q-Q plot

    dev.off()
  }

  return(results)
}

# ------------------------------
# Prepare predictors
# ------------------------------
demographic_predictors <- "Sex + Religion"
behavior_predictors <- paste(names(behaviors)[-length(names(behaviors))], collapse = " + ")
emotions_before_predictors <- paste(names(emotions_before_sh_only), collapse = " + ")
# ------------------------------
# Fit models
# ------------------------------
model_sets <- list()

# 1. Demographics predicting emotions before (no VIF needed)
model_sets$demographics_emob <- fit_robust_models(names(emotions_before_sh_only),
demographic_predictors, data_sh_only)
write_csv(model_sets$demographics_emob, "demographics_emob_results.csv")

# 2. Demographics predicting emotions after (no VIF needed)
model_sets$demographics_emoa <- fit_robust_models(names(emotions_after_sh_only),
demographic_predictors, data_sh_only)
write_csv(model_sets$demographics_emoa, "demographics_emoa_results.csv")

# 3. Demographics predicting emotion deltas (no VIF needed)
model_sets$demographics_delta <- fit_robust_models(names(delta_emotions), demographic_predictors,
delta_df)
write_csv(model_sets$demographics_delta, "demographics_delta_results.csv")

# 4. Demographics predicting behaviors (no VIF needed)
model_sets$demographics_behaviors <- fit_robust_models(names(behaviors), demographic_predictors,
data_clean)
write_csv(model_sets$demographics_behaviors, "demographics_behaviors_results.csv")

# 5. Demographics predicting behaviors in SH>0 (no VIF needed)
```

```
model_sets$demographics_behaviors_sh <- fit_robust_models(names(behaviors),
demographic_predictors, data_sh_only)
write_csv(model_sets$demographics_behaviors_sh, "demographics_behaviors_sh_results.csv")

behavior_predictors <- paste(names(behaviors_sh_only)[-length(names(behaviors_sh_only))], collapse
= " + ")

# 6. Behaviors predicting emotions after (check VIF)
model_sets$behaviors_emoa <- fit_robust_models(names(emotions_after_sh_only), behavior_predictors,
data_sh_only,
                                               check_vif = TRUE, set_name = "behaviors_emoa")
write_csv(model_sets$behaviors_emoa, "behaviors_emoa_results.csv")

# 7. Behaviors predicting emotion deltas (check VIF)
model_sets$behaviors_delta <- fit_robust_models(names(delta_emotions), behavior_predictors,
delta_df,
                                                check_vif = TRUE, set_name = "behaviors_delta")
write_csv(model_sets$behaviors_delta, "behaviors_delta_results.csv")

# 8. Emotions before predicting behaviors (check VIF)
model_sets$emob_behaviors <- fit_robust_models(names(behaviors_sh_only),
emotions_before_predictors, data_sh_only,
                                               check_vif = TRUE, set_name = "emob_behaviors")
write_csv(model_sets$emob_behaviors, "emob_behaviors_results.csv")

# ------------------------------
# ORDINAL TESTING ANGER
# ------------------------------
ordinal_data <- data_sh_only %>%
  select(ID, Sex, Religion) %>%
  left_join(
    data %>% select(ID, Anger_emob, Anger_emoa),
    by = "ID"
  )

ordinal_anger_before <- clm(
  factor(Anger_emob, ordered = TRUE) ~ Sex + Religion,
  data = data_sh_only
)

ordinal_anger_after <- clm(
  factor(Anger_emoa, ordered = TRUE) ~ Sex + Religion,
  data = data_sh_only
)

summary(ordinal_anger_before)
summary(ordinal_anger_after)

# ------------------------------
# Prepare predictors
# ------------------------------
demographic_predictors <- c(
  demographic_predictors,
  "Avg_beh"
)

fit_robust_models <- function(outcome_vars, predictors, data, check_vif = FALSE, set_name = NULL)
{

  results <- map_df(outcome_vars, function(var) {

    formula <- as.formula(
      paste(var, "~", paste(predictors, collapse = " + "))
    )
```

```
    lm_robust(formula, data = data, se_type = "HC2") %>%
      tidy() %>%
      mutate(Outcome = var)
  })

  # FDR correction
  results <- results %>%
    group_by(term) %>%
    mutate(
      p_adj = p.adjust(p.value, method = "fdr"),
      nominally_significant = p.value < 0.05,
      fdr_significant = p_adj < 0.05
    ) %>%
    ungroup()

  # VIF + simple diagnostic plots
  if (check_vif & !is.null(set_name)) {

    # VIF
    vif_model <- lm(
      as.formula(
        paste(outcome_vars[1], "~", paste(predictors, collapse = " + "))
      ),
      data = data
    )

    vif_table <- tibble(
      Predictor = names(vif(vif_model)),
      VIF = as.numeric(vif(vif_model))
    )

    write_csv(
      vif_table,
      paste0("VIF_", set_name, ".csv")
    )

    # Diagnostic plots
    png(
      paste0("Diagnostics_", set_name, ".png"),
      width = 1200,
      height = 600
    )

    par(mfrow = c(1, 2))

    plot(vif_model, which = 1)
    plot(vif_model, which = 2)

    dev.off()
  }

  return(results)
}

# ------------------------------
# Fit covariate models
# ------------------------------

# 9. Demographics predicting emotions before with COVAR
model_sets$demographics_emob_covar <- fit_robust_models(names(emotions_before_sh_only),
demographic_predictors, data_sh_only)
write_csv(model_sets$demographics_emob_covar, "demographics_covar_emob_results.csv")

# 10. Demographics predicting emotions after with COVAR
model_sets$demographics_emoa_covar <- fit_robust_models(names(emotions_after_sh_only),
```

```
demographic_predictors, data_sh_only)
write_csv(model_sets$demographics_emoa_covar, "demographics_covar_emoa_results.csv")

# 11. Demographics predicting emotion deltas with COVAR
model_sets$demographics_delta_covar <- fit_robust_models(names(delta_emotions),
demographic_predictors, delta_df)
write_csv(model_sets$demographics_delta_covar, "demographics_covar_delta_results.csv")

# ------------------------------
# ORDINAL TESTING ANGER
# ------------------------------
ordinal_data <- data_sh_only %>%
  select(ID, Sex, Religion, Avg_beh) %>%
  left_join(
    data %>% select(ID, Anger_emob, Anger_emoa),
    by = "ID"
  )

ordinal_anger_before <- clm(
  factor(Anger_emob, ordered = TRUE) ~ Sex + Religion + Avg_beh,
  data = data_sh_only
)

ordinal_anger_after <- clm(
  factor(Anger_emoa, ordered = TRUE) ~ Sex + Religion + Avg_beh,
  data = data_sh_only
)

summary(ordinal_anger_before)
summary(ordinal_anger_after)

# ------------------------------
# Save plots for all model sets
# ------------------------------
names(model_sets) %>%
  walk(~ plot_coeffs(model_sets[[.x]],
                     title = str_replace_all(.x, "_", " ") %>% str_to_title(),
                     filename = paste0(.x, ".png")))
```
